# Oral immunotherapy with the ingestion of house dust mite extract in a murine model of allergic asthma

**DOI:** 10.1186/s13223-018-0269-2

**Published:** 2018-10-16

**Authors:** Yao-Tung Wang, Hsu-Chung Liu, Hui-Chen Chen, Yen-Ching Lee, Tung-Chou Tsai, Hsiao-Ling Chen, Hueng-Chuen Fan, Chuan-Mu Chen

**Affiliations:** 1 Division of Pulmonary Medicine, Department of Internal Medicine, Chung Shan Medical University Hospital, Taichung, Taiwan; 2 School of Medicine, Chung Shan Medical University, Taichung, Taiwan; 3 Division of Chest Medicine, Department of Internal Medicine, Cheng Ching Hospital, Taichung, Taiwan; 4 Department of Life Sciences, College of Life Sciences, National Chung Hsing University, No. 250, Kuo-Kuang Road, Taichung, 402 Taiwan; 5 Department of Microbiology and Immunology, School of Medicine, China Medical University, Taichung, Taiwan; 6 Department of Bioresources, Da-Yeh University, Changhwa, Taiwan; 7 Department of Pediatrics, Tungs’ Taichung Metroharbor Hospital, No. 699, Sec. 8, Taiwan Blvd., Wuchi, Taichung, 435 Taiwan; 8 Department of Medical Research, Tungs’ Taichung Metroharbor Hospital, No. 699, Sec. 8, Taiwan Blvd., Wuchi, Taichung, 435 Taiwan; 9 Department of Rehabilitation, Jen-Teh Junior College of Medicine, Nursing and Management, Miaoli, Taiwan; 10 The iEGG and Animal Biotechnology Center, National Chung Hsing University, Taichung, Taiwan; 11 Rong Hsing Research Center for Translational Medicine, National Chung Hsing University, Taichung, Taiwan

**Keywords:** Allergen-specific immunotherapy, House dust mite, Allergic asthma, Oral immunotherapy, Airway inflammation

## Abstract

**Background:**

Allergen-specific immunotherapy (ASIT) has the potential to modify allergic diseases, and it is also considered a potential therapy for allergic asthma. House dust mite (HDM) allergens, a common source of airborne allergen in human diseases, have been developed as an immunotherapy for patients with allergic asthma via the subcutaneous and sublingual routes. Oral immunotherapy with repeated allergen ingestion is emerging as another potential modality of ASIT. The aim of this study was to evaluate the therapeutic efficacy of the oral ingestion of HDM extracts in a murine model of allergic asthma.

**Methods:**

BABL/c mice were sensitized twice by intraperitoneal injection of HDM extracts and Al(OH)_3_ on day 1 and day 8. Then, the mice received challenge to induce airway inflammation by intratracheal instillation of HDM extracts on days 29–31. The treatment group received immunotherapy with oral HDM extracts ingestion before the challenge. All the mice were sacrificed on day 32 for bronchoalveolar inflammatory cytokines, mediastinal lymph node T cells, lung histology, and serum HDM-specific immunoglobulins analyses.

**Results:**

Upon HDM sensitization and following challenge, a robust Th2 cell response and eosinophilic airway inflammation were observed in mice of the positive control group. The mice treated with HDM extracts ingestion had decreased eosinophilic airway inflammation, suppressed HDM-specific Th2 cell responses in the mediastinal lymph nodes, and attenuated serum HDM-specific IgE levels.

**Conclusions:**

Oral immunotherapy with HDM extracts ingestion was demonstrated to have a partial therapeutic effect in the murine model of allergic asthma. This study may serve as the basis for the further development of oral immunotherapy with HDM extracts in allergic asthma.

## Background

Allergic asthma, an allergic disease, is characterized by Th2 cell-mediated airway inflammation and a hypersensitive reaction to allergen exposure. Allergen-specific immunotherapy (ASIT) is the repeated administration of specific, relevant allergens to treat IgE-mediated allergic disease. It is predicted that ASIT has the potential to modify the disease course of allergic asthma [1]. In the past 100 years, many studies regarding ASIT have promoted the development of many modalities of immunotherapy in allergic diseases [2]. Because the house dust mite (HDM) is an important airborne allergen source associated with asthma attacks in the domestic environment, many ASIT studies have been conducted using HDM extracts to treat asthma. There are two major immunotherapy modalities for the clinical application of allergic asthma, subcutaneous immunotherapy (SCIT) [3] and sublingual immunotherapy (SLIT) [4].

In addition, the induction of immune tolerance through repeated ingestion of allergens, called oral immunotherapy, is a novel modality of immunotherapy [5]. Although murine models of allergic asthma have been used to analyze disease mechanisms and to develop new therapies in past decades [6, 7], there have been few animal studies evaluating ASIT with an oral administration route of the HDM allergens in allergic asthma. In a study by Hsu et al. [8], the oral administration of recombinant *Dermatophagoides pteronyssinus* allergen 5 (Dp 5), produced by plants, was demonstrated to down-regulate allergen-induced airway inflammation in mice [8]. In our previous studies, oral ingestion of transgenic milk containing recombinant *D. pteronyssinus* allergen 2 (Dp 2) was demonstrated to partially protect mice from subsequent development of allergic airway inflammation [9]. These two studies used single isolated HDM allergens as airway inflammation irritants and as the oral ingestion formula. However, the whole mite extract is a complex compound and more representative of real-life aeroallergen exposure in humans [10]. There were only few experimental asthma studies focusing on the oral ingestion of HDM extracts. The aim of this study was to evaluate the therapeutic efficacy of oral HDM extracts ingestion as an immunotherapy modality for allergic asthma in the murine model.

## Methods

### An murine model of allergic airway inflammation

Commercial HDM extracts with low endotoxin content (*D. pteronyssinus* protein 39.6 mg/vial; endotoxin 25,500 EU/vial) were used in an animal model of HDM-specific allergic airway inflammation. They were purchased from Greer Laboratories (Lenoir, North Carolina, USA). The HDM extracts were dissolved in sterile phosphate-buffered saline (PBS; 2.5 mg protein weight/mL) before being used for intraperitoneal sensitization, intratracheal challenge, and oral ingestion.

**Fig. 1:**
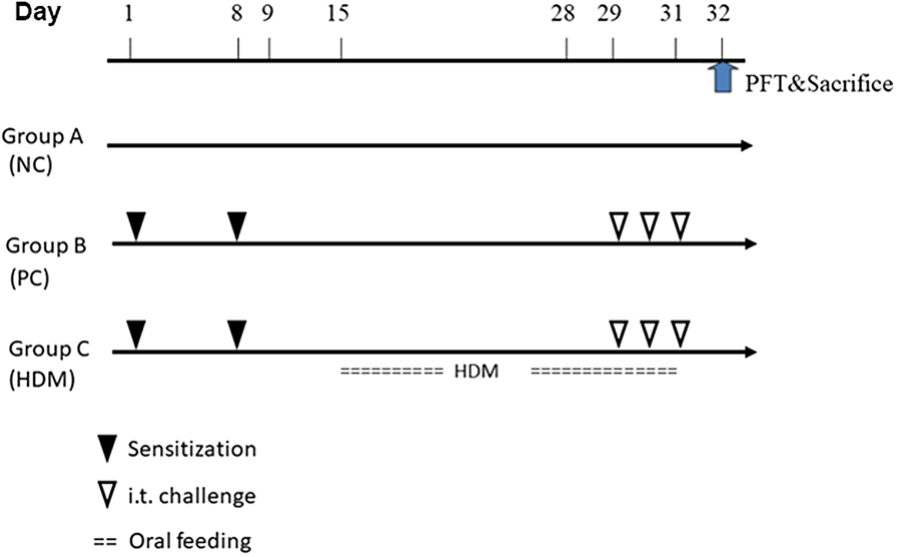
Protocol of oral immunotherapy with HDM extracts ingestion in a murine model of allergic asthma. Mice were divided into 3 groups. These mice received sensitization twice by intraperitoneal (i.p.) injection of HDM extracts and Al(OH)_3_ on day 1 and day 8 (Groups B and C). Then, the mice received allergen challenge to induce allergic airway inflammation by intratracheal (i.t.) instillation of HDM extracts on days 29–31 (Groups B and C). The mice of Group C received oral immunotherapy with HDM extracts ingestion before the allergen challenge. Group A: the normal control (NC) group composed of unsensitized mice fed formula; Group B: the positive control (PC) group composed of HDM-sensitized mice, which served as the inflammation control; Group C: the treatment (HDM) group, composed of HDM-sensitized mice treated with oral immunotherapy, which served as the intervention group

Six-week-old female BALB/c mice were obtained from the animal-breeding center of the College of Medicine, at National Taiwan University. All mice were housed under specific pathogen-free and dust mite-free conditions. The body weight of mice was controlled within a 5% variation of 25 g. The animal trials in this study were approved by the Institutional Animal Care and Use Committee of National Chung Hsing University, Taiwan (IACUC No.104-123). Initially, these mice received sensitization (intraperitoneal injection) twice on days 1 and 8 with 25 μg of HDM extracts and 2 mg of Al(OH)_3_ in 200 μL of PBS. Alum, Al(OH)_3_ (Alu-gel-S, Serva, Heidelberg, Germany), was used as an adjuvant for the promotion of T helper cell 2 (Th2) immunologic response in mice [11]. Mice were divided into three experimental groups: (A) the normal control (NC) group, composed of unsensitized mice fed formula who did not receive intraperitoneal injections of the HDM extracts; (B) the positive control (PC) group, composed of HDM-sensitized mice that received sensitization and following challenge with HDM extracts to induce allergic airway inflammation and that served as the inflammation control; and (C) the treatment (HDM) group, composed of HDM-treated mice that received HDM extracts orally daily from day 15 to day 31 and that served as the intervention group. To induce allergic airway inflammation [11–13], the mice in groups B and C received allergen challenges by intratracheal (i.t.) instillation with 100 μg HDM extracts in 50 μL of PBS under light anesthesia three times on three consecutive days (day 29–day 31). The mice of group A only received i.t. challenge with PBS. The timeline of the animal trial is shown in Fig. 1.

### Analysis of inflammatory cell counts and cytokine expression levels in BAL fluids

After i.t. challenge, the experimental mice were sacrificed for the collection of bronchoalveolar lavage (BAL) fluid at day 32. BAL fluid collection and the analysis of inflammatory cells were performed as previous reported [14]. One milliliter of Hanks’ balanced salts (HBSS) free of ionized calcium and magnesium but supplemented with 0.05 mmol/L sodium EDTA was instilled four times via the tracheal cannula and recovered by gentle manual aspiration. The recovered fluids were centrifuged (700*g* for 10 min at 4 °C). The cell pellets were washed twice and finally resuspended in 1 mL of HBSS. The percentage of leukocytes among the BAL cells was determined with a hemocytometer (VWR, Lutterworth, UK). The cytospin preparation of 100 μL of BAL fluids was followed by staining with Liu stain. The differential counts of BAL cells (eosinophil, neutrophil, lymphocyte) were performed under a microscope, and 200 total cells were counted. In addition, the supernatants of the BAL fluid were aspirated and stored at − 80 °C until the detection of the cytokine levels. The analysis of the cytokine levels in the BAL fluids was done via paired antibodies for murine IFN-γ, IL-4, and IL-5 in standardized sandwich ELISAs according to the manufacturer’s protocol [15].

### Cytokine assays of mediastinal lymph node T cells after HDM re-stimulation

After BAL, the mediastinal lymph nodes were dissected and placed separately into 4 °C HBSS. The mediastinal lymph nodes were digested with collagenase I (Gibco Invitrogen, Grand Island, NY, USA) and DNase (Roche Applied Science, Mannheim, Germany) for 30 min at 37 °C. We filtered the cell suspensions through a 70-μm cell strainer and depleted them of red blood cells using red blood cell lysis buffer. The recovered cells were filtered through a 70-μm nylon sieve (BD Falcon, San Jose, CA, USA), washed twice, resuspended in complete media, and counted with a hemocytometer. We pooled cells from each group and cultured 1.5 × 10^6^ lymph node cells in triplicate for 72 h in medium with or without HDM extracts (100 μg/mL) in 96-well plates, respectively. The culture medium was RPMI 1640 (Perbio, Waltham, MA, USA) containing 5% heat-inactivated fetal calf serum (FCS), 50 mM 2-mercaptoethanol and penicillin–streptomycin (Gibco Invitrogen, Grand Island, NY, USA) [16]. The supernatants were analyzed for the expression levels of IFN-γ (Th1 cytokine) and IL-5 (Th2 cytokine) by ELISAs [17].

### Lung tissue histology and scoring of pulmonary inflammation

The left lungs were fixed in formalin. Paraffin-embedded sections were stained with hematoxylin and eosin (H&E) for histological analysis and comparison. Pieces from all lung lobes are used for histological examination. Sections of 2.5-μm thickness are cut and stained with H&E [18, 19]. A scoring and grading method of lung inflammation was used as in previous studies [14]. A value from 0 to 3 criterion was scored for each tissue section. Two separate scoring criteria were documented for pulmonary inflammation: peribronchial inflammation and perivascular inflammation. A value of 0 was assigned when no inflammation was detectable, a value of 1 was assigned when occasional cuffing with inflammatory cells was observed, a value of 2 was assigned when most bronchi or vessels were surrounded by a thin layer (one to five cells) of inflammatory cells, and a value of 3 was assigned when most bronchi or vessels were surrounded by a thick layer (more than 5 cells) of inflammatory cells. In total, 10 tissue sections per mouse were scored in high power fields (×400), and the inflammation scores were expressed mean values and compared between groups.

### Measurements of serum HDM-specific immunoglobulins

Blood was collected from the hearts of the mice after they were sacrificed. The blood was centrifuged at 2500 rpm for 20 min. The serum was collected and stored at − 80 °C before analysis. The serum HDM-specific IgE, IgG1, and IgG2a levels were determined by using ELISA kits, and the measurements were done according to the manufacture’s protocol [20].

### Analysis of regulatory T (Treg) cell populations

We pooled the cells from the mediastinal lymph nodes in each group and cultured 4 × 10^6^ T cells in 96-well plates [16]. For intracellular staining, we incubated cells with 100 μg/mL HDM extracts and 1 μg/mL anti-CD28 antibody (Becton–Dickinson, East Rutherford, NJ, USA) for 6 h. Brefeldin A (5 mg/mL; Sigma-Aldrich, St. Louis, MO, USA) was added during the last 4 h. Then, we stained the cells with a monoclonal antibody (mAb) against CD4, fixed the cells, permeabilized them with the Cytofix/Cytoperm reagent (Becton–Dickinson, East Rutherford, NJ, USA), and stained them with an antibody against CD40L (Becton–Dickinson, East Rutherford, NJ, USA) and mAb against Foxp3 (eBioscience, San Diego, CA, USA). Finally, we analyzed them by fluorescence activated cell sorting (FACS; Beckman Coulter Inc., Brea, CA, USA).

### Statistical analysis

To assess the different levels of airway inflammation, a repeated measure ANOVA was performed to compare between groups. A value of *P *< 0.05 was used to indicate statistical significance.

## Results

### Therapeutic effect of HDM extracts ingestion on allergic airway inflammation

**Fig. 2:**
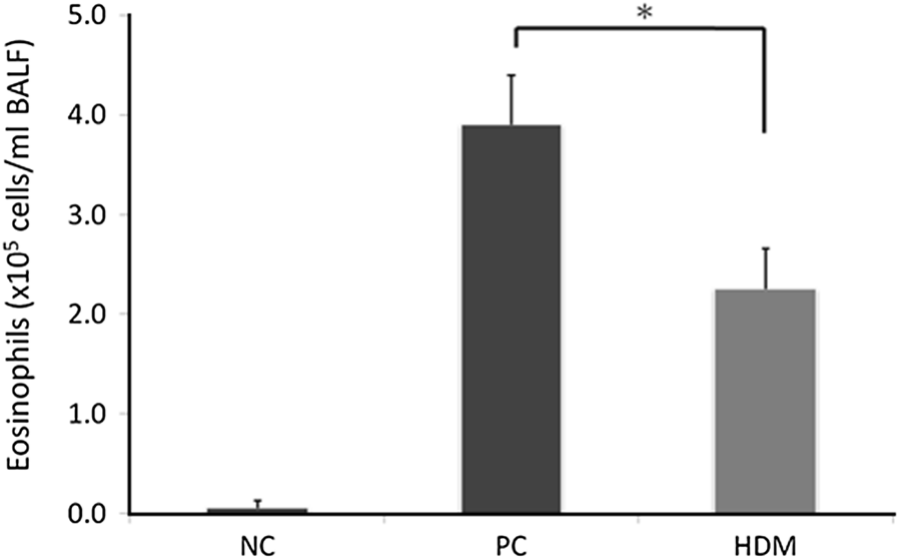
Eosinophil cell count in the bronchoalveolar lavage fluid (BALF) of different mice groups. Allergic airway inflammation was measured as the eosinophil cell counts of the BALF. Eosinophil cells were counted by cytospin and microscopy (n = 5–6). *P < 0.05

**Fig. 3:**
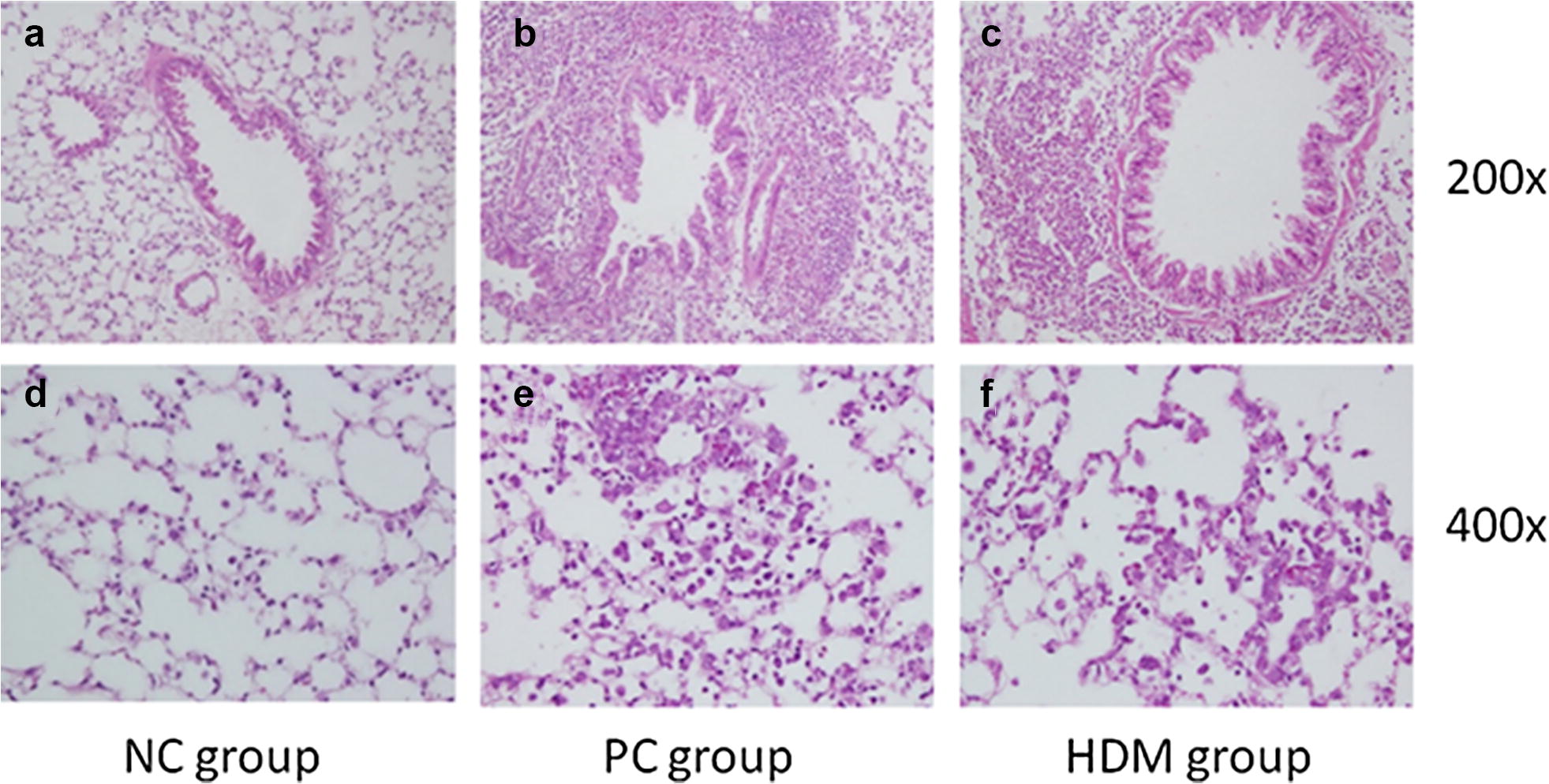
Histological analysis of allergic airway inflammation by immunohistochemical (IHC) staining. **a**, **d** Image representative of the negative control from normal mice. **b**, **e** Image representative of the positive control from the HDM-sensitized mice that received following challenge with HDM extractrs. **c**, **f** Image representative of the HDM group that HDM-sensitized mice received oral immunotherapy with HDM extracts ingestion before the challenge. There were numerous inflammatory cells that had infiltrated beneath the tracheal epithelium in the PC and HDM groups. Representative photomicrographs of different groups are shown; n = 5 mice per group

**Fig. 4:**
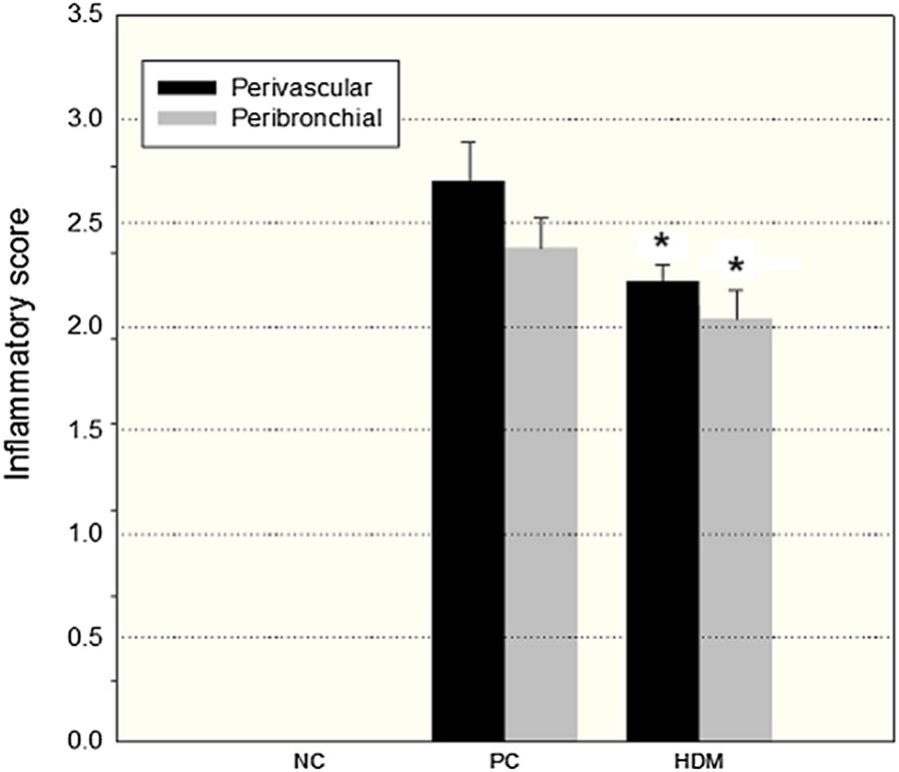
The scoring of pulmonary inflammation in lung histology. The degrees of pulmonary inflammation observed in these mice were defined as the average value of the peribronchial and perivascular inflammatory scores. (n = 5) *P < 0.05, compared to PC group

To determine the therapeutic effect of HDM extracts on the inflammatory response, the differential cell counts in the BAL fluids of mice were measured to evaluate the extent of airway inflammation. The HDM group had fewer eosinophil cells in BAL fluids than PC group (Fig. 2). The PC group had obvious inflammatory cell infiltration in both bronchi (Fig. 3b) and the lung parenchyma (Fig. 3e). However, the HDM group had less infiltration of inflammatory cells (Figs. 3c, f) than the PC group. Furthermore, the peribronchial and perivascular pulmonary inflammation were scored according to the inflammatory scoring criteria. Both the peribronchial and perivascular inflammatory scores of the HDM group were lower than those of the PC group (Fig. 4).

These findings suggest that oral immunotherapy the ingestion of HDM extracts could suppress eosinophilic airway inflammation in mice with established HDM-specific sensitization and following a challenge.

**Fig. 5:**
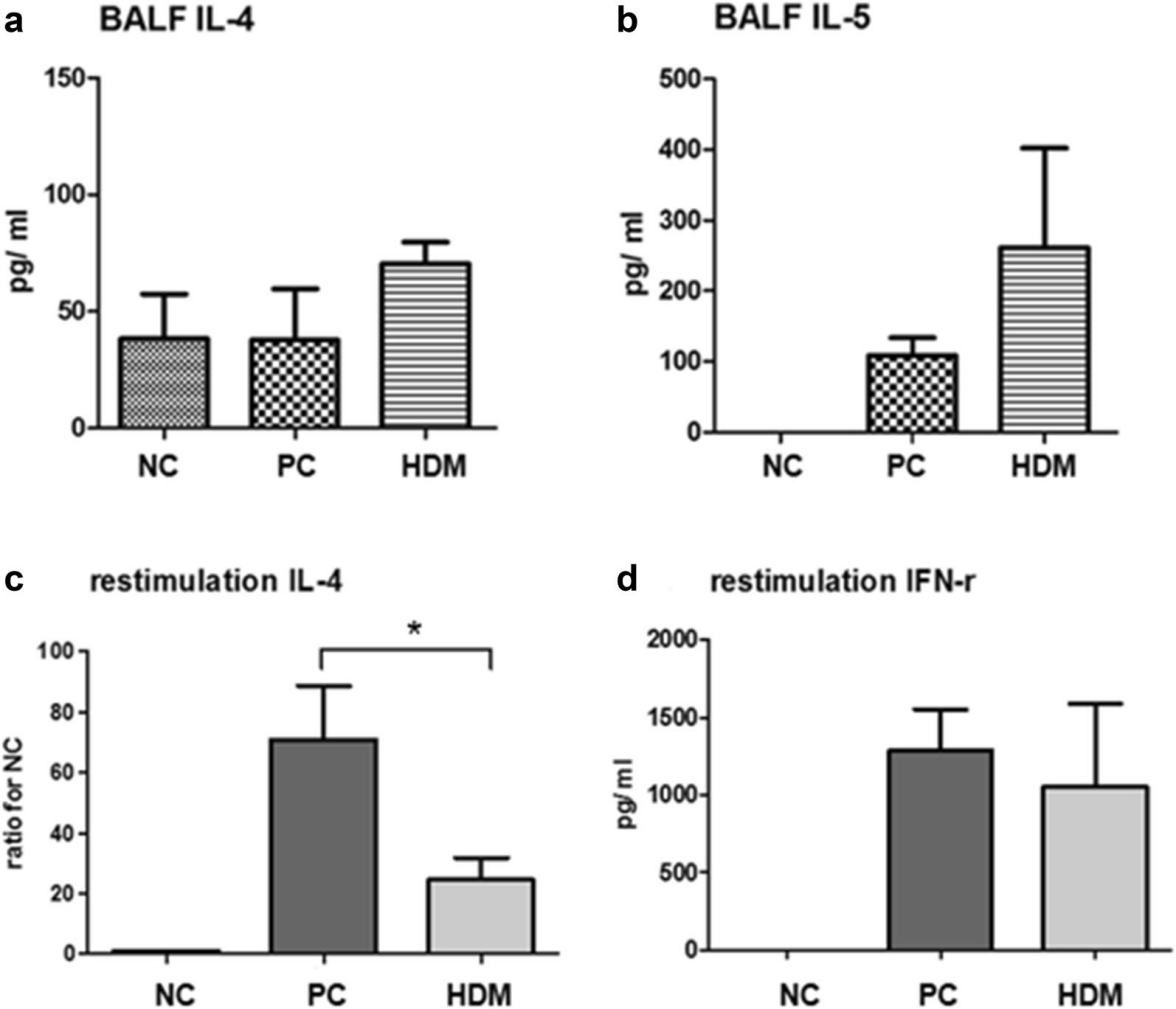
Inflammatory cytokine levels and T cell response. **a**, **b** IL-4 and IL-5 levels in BAL fluids. **c**, **d** IL-4 and INF-γ secretion levels of the mediastinal lymph node T cells after re-stimulation with HDM extracts for 72 h. *P < 0.05

The Th2 cytokine levels, such as IL-4 and IL-5, were also measured in these BAL fluids. The results showed that there were no significant differences between the HDM group and the PC group (Figs. 5a, b). The Th1 cytokine level of INF-γ was also tested in the BAL fluids, but the levels were undetectable in all three groups (data not shown).

### Effect of HDM extracts ingestion on T cell function of the lymph nodes

The mediastinal lymph node T cells were collected from the individual mice. These cells were co-cultured in triplicate with 100 μg/mL HDM extracts for a reaction of re-stimulation. The supernatants were analyzed for the levels of IL-4 (Th2 cytokine) and INF-γ (Th1 cytokine) 72 h later. The results showed that the HDM group had a significantly lower IL-4 level than the PC group (Fig. 5c). However, the INF-γ level in the HDM group was not significantly different than that of the PC group (Fig. 5d). These findings suggested that the ingestion of HDM extracts could suppress the HDM-specific response of Th2 cells in the mediastinal lymph nodes.

### Therapeutic effect of HDM extracts ingestion on serum HDM-specific IgE

**Fig. 6:**
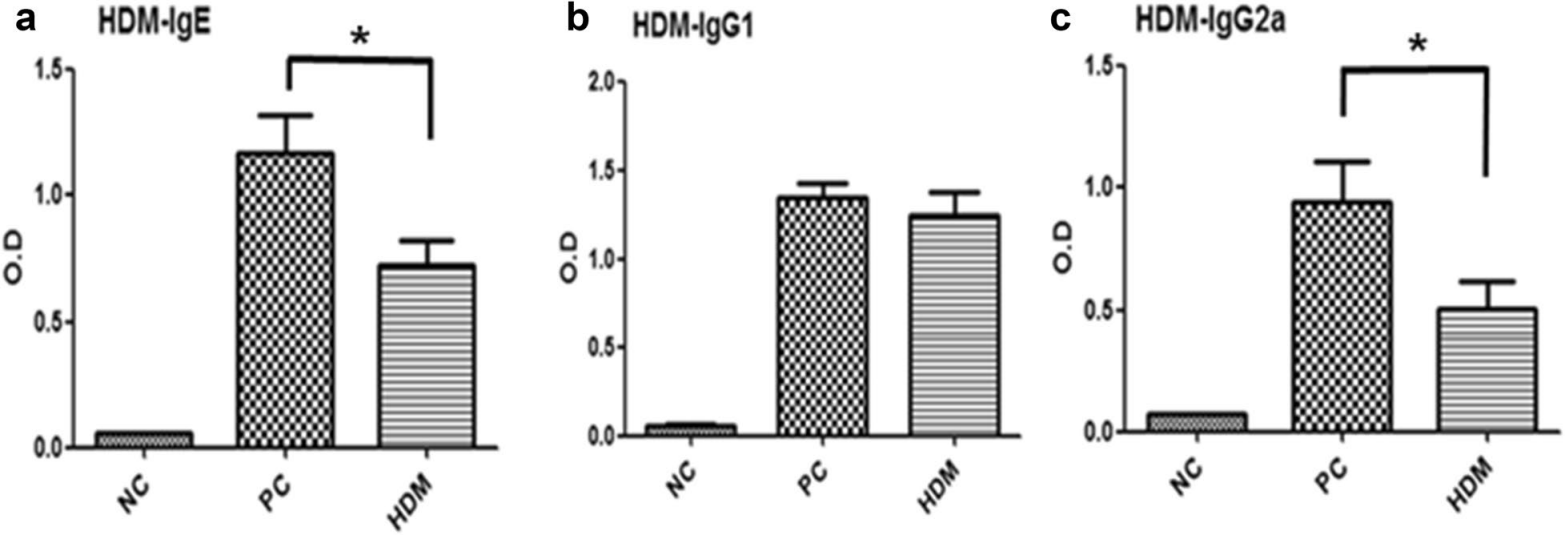
Serum HDM-specific immunoglobulin levels. Serum samples from mice in the three experimental groups were analyzed to determine the HDM-specific IgE levels (**a**), HDM-specific IgG1 levels (**b**), and HDM-specific IgG2a levels (**c**) by ELISA. The data are expressed as the mean ± SD of the values obtained for the individual mice. n = 5–6 mice per group. *P < 0.05

The serum levels of HDM-specific immunoglobulin were analyzed by ELISA. The HDM group had lower serum HDM-specific IgE and IgG2a levels than the PC group (Fig. 6). There was no statistical difference in the level of HDM-specific IgG1 between the HDM group and the PC group. These data suggested that the ingestion of HDM extracts could suppress the Th2 response and, subsequently, the HDM-specific IgE level in these HDM-sensitized mice.

### The regulatory T cells (Treg) population from the mediastinal lymph nodes

**Fig. 7:**
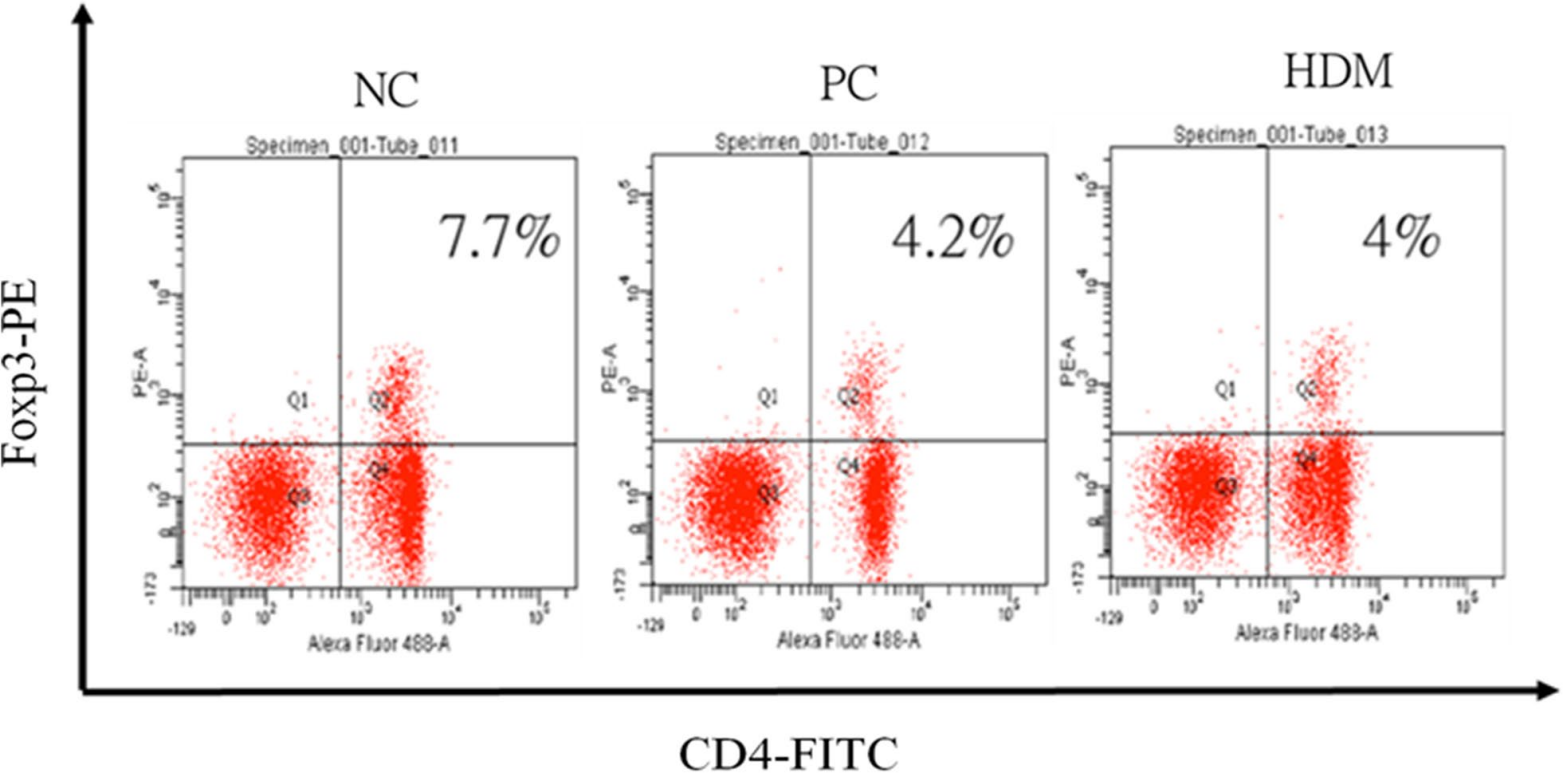
The regulatory T cells (Treg) population from the mediastinal lymph nodes. Flow cytometry analysis of the percentage of Treg cells are shown as both positive staining for CD4 and Foxp3

The mediastinal lymph node cells were collected and pooled in vitro with HDM extracts (100 μg/mL). After 24 h of HDM extracts stimulation, the cells were stained for CD4 and Foxp3. The percentage of CD4+Foxp3+ Treg cells in the CD4+ T cell population from the three experimental groups are shown in the upper right quadrant of Fig. 7. The results showed that there was no significant increase in the Treg cell percentage in the HDM group compared with the PC group. This finding suggested that the therapeutic effect of HDM extracts ingestion in this model may not be directly mediated by the up-regulation of Treg cells.

## Discussion

In this murine study of allergic asthma, the intra-peritoneally HDM-sensitized mice developed eosinophilic airway inflammation after the intra-tracheal challenge with HDM extracts. The oral ingestion of HDM extracts was demonstrated to have a therapeutic effect; it decreased eosinophilic airway inflammation, suppressed the HDM-specific Th2 cell response in the mediastinal lymph node, and attenuated the serum HDM-specific IgE level. However, the histological and bronchoalveolar lavage analysis showed that the HDM group still had an obvious inflammatory reaction when compared with the NC group. This result reveals that the allergic airway inflammation seems to be not completely resolved by this oral immunotherapy. The commercial HDM extracts used in this study are composed of various allergens and non-allergenic peptides from *D. pteronyssinus.* Some of the components have been proven to have intrinsic enzymatic activity or to have ability to induce the inflammatory response [6, 21], which implies that the exposure to HDM extracts in the airway could induce a complex Th1/Th2 inflammatory response. The confounding factor of intrinsic enzymatic activity may be one explanation for the remaining obvious inflammatory infiltrates in the histologic analysis and elevated cytokine level in the bronchoalveolar lavage in the HDM group.

Several possible underlying mechanisms of ASIT have been proposed, including tachyphylaxis, the induction of T cell anergy, switching from a Th2 to a Th1 response, and Treg cells [2]. It has been proposed that the shift from an allergen-specific effector T cell response to a Treg cell response appears to be a key event in the induction of tolerance in patients undergoing ASIT [22]. However, our analysis of the Treg cell population in the mediastinal lymph nodes showed no significant up-regulation of Treg cells in mice received treatment with HDM extracts ingestion. The absence of up-regulated Treg cells in this study may be related to the treatment dose of the HDM extracts. It is thought that low doses of antigen ingestion favor tolerance driven by Treg cells, whereas high doses of antigen ingestion favor tolerance mediated by lymphocyte anergy [5]. Therefore, anergy-driven tolerance could be a more possible mechanism to explain the therapeutic effect of oral immunotherapy with HDM extracts ingestion in this study.

From clinical studies in patients with asthma and HDM allergy, both subcutaneous immunotherapy or sublingual immunotherapy with HDM extracts have been demonstrated to have therapeutic effects on reducing symptoms and the use of medication [3, 23]. Although there is a mild to moderate potential benefit, the concern of the risk of adverse effects and the inconvenience in receiving prolonged course of immunotherapy impede patient preferences for this treatment modality. Therefore, ASIT is considered to have weak evidence and limited application in clinical asthma management guideline [24]. In comparison to the subcutaneous and sublingual routes of allergen administration, oral allergen ingestion has the advantage of being more convenient in practice. It has been well established from previous studies in both animals and humans that repeated allergen exposure in the gut mucosa can induce immune tolerance [5, 25]. This study demonstrated that oral immunotherapy with HDM extracts ingestion could be a potential alternative immunotherapy modality in treating allergic asthma. However, further clinical studies are needed to compare the efficacy and safety among oral immunotherapy and the relative well-established models of SCIT and SLIT.

It is well known that mice could not spontaneously develop allergic asthma. Several allergen challenge models in mice have been developed for studying asthma and they could reproduce airway inflammatory features mimicking allergic asthma. Our sensitization and challenge protocol using intraperitoneal allergen injection and intratracheal instillation was according to the basic rules of acute allergen challenge models described in previous studies [6, 7]. A successful sensitization usually requires multiple systemic administration of target allergen with the coincidences of adjuvant. Aluminum hydroxide Al(OH)_3_ are common adjuvant used to promote a Th2 phenotype inflammation in mice when they are mixed with antigen via intraperitoneal administration. Because asthma is defined as a chronic inflammatory disease of airways, murine experiments of allergen challenge models have some disparity indeed both in immunology and anatomy when comparing with clinical asthma. The findings in this acute allergen challenge model still have limitations when extrapolating the results to the human disease.

## Conclusions

In summary, oral immunotherapy with HDM extracts ingestion in the murine model of allergic asthma demonstrated a partial therapeutic effect. This study provides helpful information on the further development of oral immunotherapy in allergic asthma.

## Abbreviations

ASIT: allergen-specific immunotherapy
BAL: bronchoalveolar lavage
Dp2: group 2 allergen from *Dermatophagoides pteronyssinus*
Dp5: group 5 allergen from *Dermatophagoides pteronyssinus*
ELISA: enzyme-linked immunosorbent assay
HBSS: Hanks’ balanced salts
HDMs: house dust mites
H&E: hematoxylin and eosin
SCIT: subcutaneous injection immunotherapy
SLIT: sublingual immunotherapy
Th2: T helper cell 2
Treg: regulatory T cells
